# Pregnant People-Infant Linkage for Surveillance

**DOI:** 10.23889/ijpds.v9i5.2636

**Published:** 2024-09-18

**Authors:** Anna Ferrante, Adrian Brown, Sean Randall, James Boyd

**Affiliations:** 1 Curtin University; 2 Deakin University; 3 Deakin University

## Introduction

Organisations are increasingly aware of the risks and responsibilities of handling personally identifying information (PII). These factors not only influence internal data management practices but also impact on data linkage arrangements with other parties. Some linkage environments are particularly sensitive to the release or use of PII. Advances in privacy-preserving record linkage methods such as PPRL-using-Bloom make it possible to undertake highly accurate data linkage without release or disclosure of PII. Such methods play a role in enabling data linkage in risk-sensitive environments.

## Objectives and Approach

We present and describe several Australian case studies where the PPRL-using-Bloom method has been used to enable data linkage between organisations. We report on the defining elements of each case, the associated risks and solutions, as well as quality and performance issues. We also reflect on challenges and opportunities for future improvement.

## Results

Australian use cases utilising privacy preserving linkage (PPRL-using-Bloom) include projects linking state-based datasets to Commonwealth datasets, some linking primary care data to state-based secondary healthcare data, and others linking healthcare data to non-health datasets such as police and criminal justice datasets.

## Conclusion / Implications

Methods such as PPRL-using-Bloom play a critical role in enabling data linkage in highly risk-sensitive environments. However, in an ever evolving world where risks and requirements are constantly changing, linkage methodologies and technologies must remain adaptable to meet evolving demands.

